# High-Fat Diet, Epigenetics, and Atherosclerosis: A Narrative Review

**DOI:** 10.3390/nu17010127

**Published:** 2024-12-31

**Authors:** Vikrant Rai

**Affiliations:** Department of Translational Research, Western University of Health Sciences, Pomona, CA 91766, USA; vrai@westernu.edu; Tel.: +1-9094697043

**Keywords:** high-fat diet, saturated fatty acids, epigenetics, atherosclerosis, epigenetic reprogramming, therapeutics

## Abstract

Background/Objectives: Atherosclerosis is a chronic inflammatory disease developing and progressing in the presence of risk factors including hyperlipidemia, hypercholesterolemia, and chronic inflammation, among others. Atherosclerosis commonly precipitates as ischemic events, transient ischemic attacks, and myocardial infarction. Saturated fatty acids are risk factors; however, their association with epigenetics in the pathophysiology of atherosclerosis is not clearly understood. The preclinical and clinical trials associating atherosclerosis with epigenetics are scarcely documented, and most of the studies reported the use of drugs inhibiting methylation and histone modification to improve atherosclerosis. This narrative review aims to discuss various aspects and the association between a high-fat diet, epigenetic reprogramming, and atherosclerosis. Methods: A literature search with the keywords high-fat diet, epigenetics, and atherosclerosis, alone or in combination, was conducted to search for articles in the English language. Duplicate articles were removed, and articles related to the subject of this review article were included in this review. Results: A review of the literature suggests that a high-fat diet with saturated fatty acids is a risk factor for atherosclerosis, but this association is multifactorial, and epigenetics play a critical role. However, the connecting link and the underlying molecular and cellular mechanisms are not clearly understood yet and warrant more research. Conclusions: A high-fat diet rich in saturated fatty acids is a risk factor for atherosclerosis involving epigenetic reprogramming and altered gene expression. The existing preclinical and clinical trials support the role of epigenetics and reversing it using drugs to attenuate atherosclerosis, but definitive evidence warrants larger clinical trials. Further, a high-fat diet in pregnant mothers can manifest as cardiovascular disease in offspring; caution must be taken in pregnant mothers for their diet and nutrients.

## 1. Introduction

High-carbohydrate and high-fat diets are energy-rich diets affecting metabolic health and contribute to insulin resistance, obesity, diabetes, and other metabolic alterations in lipid and carbohydrate metabolism. Genetic variation, epigenetics, and gene–environment interactions in the presence of a sedentary lifestyle and stress contribute to chronic inflammatory diseases including obesity and diabetes, the risk factors for cardiovascular disease [1]. Various studies have suggested that high-saturated-fat diets (HFDs) are associated with an increased risk of cardiovascular diseases and diabetes involving insulin signaling pathways and pro-inflammatory cytokines [2]. The increased prevalence of these diseases is due to cardio-metabolic effects regulated by various genes whose expression is subject to genetic and non-genetic regulation. This non-genetic regulation is called epigenetics, the nonheritable changes in gene expression, which are the result of changes in lifestyle, altered diet (Western diet, high-fat–high-cholesterol diet, decreased vegetables, and high-glycemic-index diet), stress, and environment and are due to epigenetics which involve processes like DNA methylation, histone modification via acetylation, methylation, ubiquitylation, or phosphorylation and noncoding RNA (ncRNA)-associated gene silencing. Sumoylation, citrullination, proline isomerization, butyrylation, propionylation, ADP-ribosylation, and glycosylation are other processes involved in histone modification. ncRNAs such as microRNAs (miRNA) and small interfering RNAs (siRNA) regulate gene expression at the transcription, translational, post-transcription, and post-translational levels [1,3]. These epigenetic changes are regulated by various enzymes including DNA methyltransferases, histone methyltransferases, and histone deacetylases [4]. A high-fat diet (HFD), a risk factor for cardiovascular disease [2] and atherosclerosis [5,6], also induces epigenetic changes [7,8]. The literature associates HFDs, epigenetics, and cardiovascular diseases in general, but an exact association between HFDs, epigenetics, and atherosclerosis is not comprehensively discussed. This review aims to discuss various aspects, establishing the association between HFDs, epigenetics, and atherosclerosis.

## 2. High-Fat Diet and Atherosclerosis

**Figure 1:**
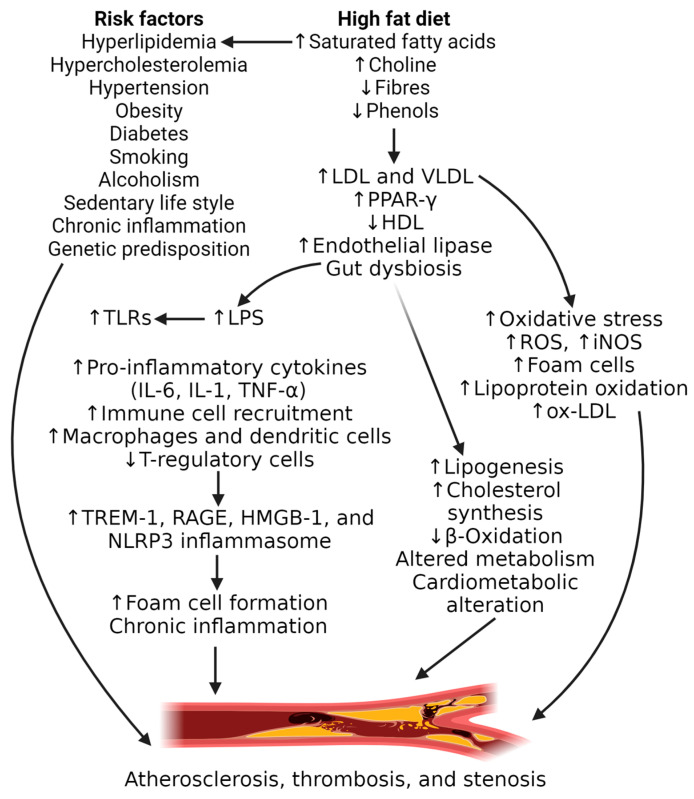
A high-fat diet is a risk factor for atherosclerosis. A high-fat diet with saturated fatty acids (SFAs) results in increased choline and endothelial lipase and decreased phenols and fibers, resulting in decreased beta-oxidation, increased lipogenesis, cholesterol synthesis, low-density lipoproteins (LDLs), very low density lipoproteins (VLDLs), and peroxisome proliferator-activated receptor gamma (PPAR-γ), and decreased high-density lipoproteins (HDLs). Increased LDL and VLDL levels lead to increased oxidative stress and reactive oxygen species (ROS), the induction of nitric oxide synthase (iNOS), oxidation of LDL, the formation of oxidized-LDL (ox-LDL), and foam cell formation. SFA accumulation also leads to gut dysbiosis resulting in increased lipopolysaccharides (LPSs), leading to the activation of mediators of inflammation including toll-like receptors (TLRs), triggering receptors expressed on myeloid cells (TREM)-1, the receptor for advanced glycation end-products (RAGE), the high-mobility group box protein (HMGB)-1, interleukins (ILs), tumor necrosis factor (TNF)-α, NOD-, LRR-, and pyrin domain-containing protein 3 (NLRP3) inflammasomes, and the recruitment of inflammatory immune cells. All these factors lead to the development and progression of atherosclerosis.

Atherosclerosis is a chronic inflammatory disease developing in the presence of risk factors such as obesity, diabetes, hyperlipidemia, hypertension, hypercholesterolemia, alcoholism, smoking, and the presence of other chronic inflammatory diseases with systemic inflammation (Figure 1).

Atherosclerosis starts as a fatty streak, and plaque builds up with the deposition of low-density lipoproteins within the intima, leading to the formation of atheromatous plaque which may be stable or unstable. Decreased vascular smooth muscle cell proliferation, increased neoangiogenesis within the plaque, intraplaque hemorrhage, chronic inflammation, and thinning of the fibrous cap make plaques prone to becoming vulnerable plaques [9,10,11,12,13,14]. Nutrients play a critical role in increasing the risk of developing atherosclerosis, and the intake of saturated fatty acids (SFAs) while eating a high-fat diet (HFD) is a common risk factor (Figure 1). The intake of SFAs should be less than 10% of total calorie intake, as suggested by the 2020–2025 Dietary Guidelines for Americans (DGA), while no limit has been suggested for total fat intake. This suggests replacing the SFAs with unsaturated fat to decrease the risk of atherosclerosis and cardiovascular events by decreasing circulatory cholesterol. This risk with SFAs is also dependent on various other factors including age, race, sex, body composition, and metabolic status and response [15]. However, inconsistency in results for establishing an association between an SFA diet and increased intima-medial thickness suggests that not only the SFA but also other factors affect the outcome [16,17]. Laguzzi et al. investigated the association between SFAs and single genetic variants and concluded that there was no evidence between the single genetic variant and subclinical atherosclerosis in the presence of a high intake of SFAs [16]. However, the evidence of the development of and increased atherosclerosis load in mice, nonhuman primates, and swine and decreased atherosclerosis with a low-fat diet in humans suggest that an HFD rich in SFAs is a risk factor for atherosclerosis [14,18,19,20]. This notion is supported by the findings of a recent study that a diet rich in SFAs and choline increases the risk of atherosclerosis by increasing the number of metabolites contributing to plaque buildup in the arteries [6]. This notion is further supported by the findings of the increased expression of endothelial lipase and inflammatory markers in macrophages in the aorta with an HFD, increasing the risk of atherosclerosis involving PPAR-γ [21].

## 3. High-Fat-Diet-Induced Epigenetic Changes

**Figure 2:**
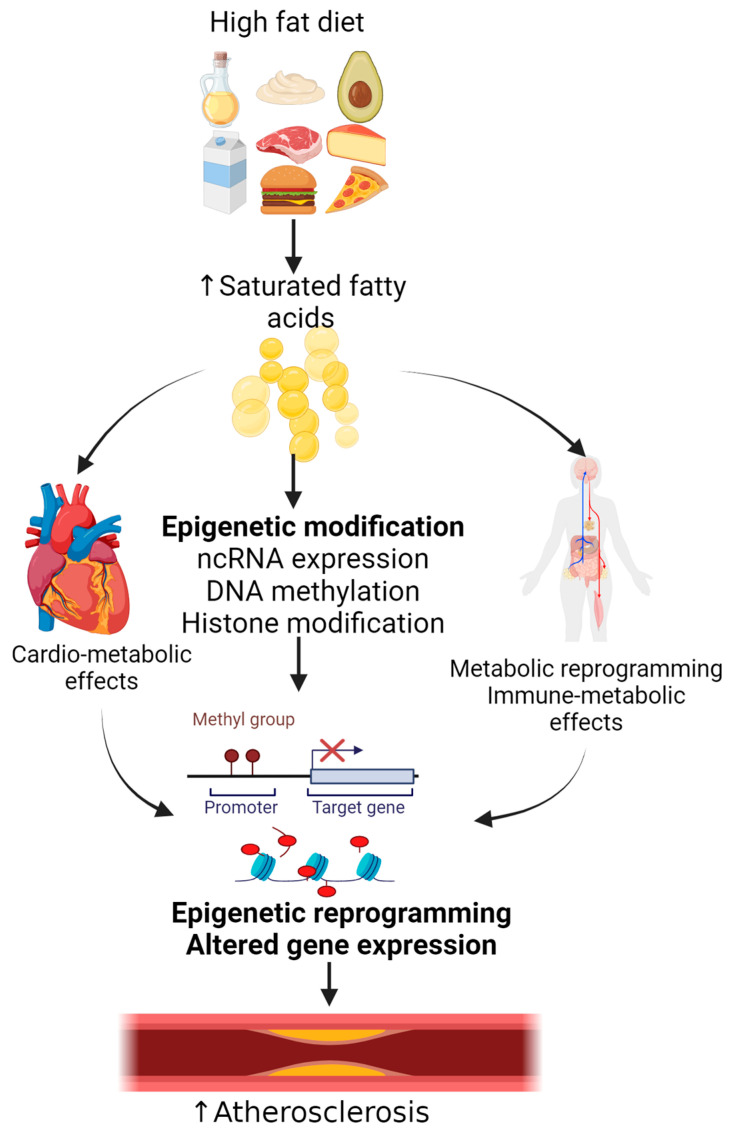
High-fat diet rich in saturated fatty acids induces epigenetic reprogramming contributing to atherosclerosis. High-fat diets induce epigenetic modification/reprogramming involving DNA methylation, histone modification, and ncRNA, changing gene expression and increasing risk of atherosclerosis.

A high-fat diet (HFD) can induce epigenetic changes that contribute to the development of various metabolic diseases. An HFD causes epigenetic dysregulation that influences gene transcription and phenotype-changing gene expression [1]. A prolonged high-fed diet induces gradual and fat-depot-specific DNA methylation changes in white adipose tissue near metabolism-related genes regulating and manipulating energy homeostatic, altered signaling, hormone secretion, and inflammation [1,22] (Figure 2). The epigenetic changes are due to the exposure to palmitate, vitamins B12 and B6, choline, methionine, folate, S-adenosylmethionine (SAM) and its product S-adenosylhomocysteine (SAH), the SAM/SAH ratio, B12, and homocysteine levels, all playing a role in altered gene expression and metabolisms. A high-fat diet alters DNA methylation and expression of an epigenetically regulated gene, SET domain-containing 2 (*SETD2*), which also responds to environmental stress. Wei et al. reported that parents fed an HFD exhibited disruption of the *SETD2* methylation pattern and perturbed SETD2 expression in the sperm of F0 and F1 mice [23]. The function of epigenetic regulators can be altered due to the metabolic stress caused by a HFD, and this leads to abnormal gene transcription causing congenital abnormalities. For example, an HFD fed to mothers results in non-compaction cardiomyopathy accompanied by decreased 5-hydroxymethylcytosine levels and altered chromatin accessibility in embryonic hearts due to metabolic changes in pregnant mice [24].

The epigenetic changes occurring during pregnancy in mothers on an HFD can be passed on to offspring with a predisposition for the development of obesity, insulin resistance, type II diabetes, and other metabolic disorders during life mainly due to DNA methylation in the pancreatic tissue and altered protein expression [25]. Zhang et al. [26], using female C57BL6/J mice fed with an HFD, reported that the HFD induces DNA methylation changes contributing to glucose intolerance in offspring involving *Irs2* and *Map2k4* gene methylation in the offspring’s liver. Another study reported that an HFD administered to pregnant mothers can disrupt one-carbon metabolism contributing to nonalcoholic fatty liver disease in offspring mice [27]. Van de Pette et al. reported that mice mothers fed with an HFD exhibit alterations in the transcriptional profile of oocytes in utero, and it ultimately leads to deregulated and ectopic expression of *Dlk1*, a prototypic paternally expressed gene, in F2 offspring born to exposed F1 mothers involving increased DNA methylation at the somatic differentially methylated region [28]. These studies suggest that an HFD administered to mothers induces epigenetic changes in utero which are passed to the offspring and that epigenetic changes are heritable and are caused by DNA methylation, histone modifications, and noncoding RNAs. It is noteworthy that epigenetic changes occurring during a lifetime are nonheritable; however, changes occurring in utero are heritable. This notion is supported by the findings of Deshpande et al. [29,30], reporting that HFD-induced epigenetic changes are heritable and diet-induced, and genetically inherited obesity contributes to differential effects on the DNA methylation in the male germline.

Metabolic reprogramming itself is not technically “epigenetic”; however, it is considered to be closely linked and deeply intertwined with epigenetic mechanisms because the changes in metabolism can directly influence epigenetic modifications through DNA methylation and histone modifications and vice versa [31]. This suggests that metabolic reprogramming induced by an HFD may induce epigenetic changes and may be of significance in atherosclerosis. An HFD suppresses levels of ATP citrate-lyase which is involved in the production of acetyl-CoA in the nucleus and cytosol and regulation of histone acetylation levels. Carrer et al. [32], using a mouse model, reported that an HFD reduces the levels of acetyl-CoA and/or the acetyl-CoA:CoA ratio in white adipose tissue, liver, and the pancreas, and acetylation of the specific histone lysine in white adipose tissue but not in the liver correlates with acetyl-CoA abundance. Acetyl-Co-A, a building block for lipids, ketone bodies, and amino acid synthesis is generated by the degradation of the amino acids including leucine, isoleucine, and tryptophan and is involved in the breakdown of carbohydrates and fatty acids through glycolysis and β-oxidation. Citrate and ATP-citrate lyase are enzymes in the Kreb’s cycle and are a source of acetyl-CoA for histone acetylation [33]. Acetyl-CoA is produced by fatty acids breaking down through beta-oxidation and directly entering the Krebs cycle, producing intermediates like succinate and alpha-ketoglutarate. Since an HFD affects levels of acetyl-CoA, it may affect the levels of succinate and alpha-ketoglutarate. This may influence histone succinylation and the activity of demethylases and hydroxylases due to their role as co-substrates in these enzymatic reactions. Increased succinate levels may inhibit enzymes that are required for alpha-ketoglutarate as a co-substrate, like histone demethylases, which remove methyl groups from histones, thereby affecting gene expression. Changing levels of alpha-ketoglutarate can influence the activity of various enzymes involved in oxygen sensing and metabolic regulation. Succinyl-CoA, which is derived from succinate, modifies histone proteins through succinylation which can alter chromatin structure and gene expression [33,34,35]. HFDs increasing acetyl-CoA production potentially may cause fluctuations in Krebs cycle intermediates which may have significant epigenetic effects via histone modifications, potentially impacting cell function [33,34,35,36]. HIF-1α activation is promoted by succinate via the inhibition of regulatory HIF hydroxylases, while it is prevented by increased long-chain fatty acids in the presence of hypoxia [37]. This suggests that an HFD may affect metabolic reprogramming, which in turn affects epigenetics in atherosclerosis, but this must be investigated in the context of atherosclerosis.

An HFD results in increased production of ketone bodies like beta-hydroxybutyrate from excess fat which can then act as a signaling molecule to influence gene expression by modifying histones through histone post-translational modification, a key mechanism linking diet to epigenetic changes. Beta-hydroxybutyrate functions as a signaling molecule by interacting with cellular receptors and influencing gene expression via beta-hydroxybutyrylation, which occurs when beta-hydroxybutyrate attaches to specific lysine residues on histone tails, altering the chromatin structure and potentially affecting gene transcription. By modifying histone proteins, beta-hydroxybutyrate may influence gene expression without changing the DNA sequence, contributing to epigenetic regulation and altering cellular functions like metabolism, the stress response, and cell differentiation [38,39,40]. It is noteworthy that excessive ketone bodies can potentially promote atherosclerosis by increasing inflammation within the blood vessels through increased production of inflammatory mediators. This leads to endothelial dysfunction, promoting the adhesion of white blood cells to the vessel wall, a crucial step in plaque formation. High ketone levels also contribute to increased reactive oxygen species production, further promoting inflammation and damaged vascular lining via endothelial damage and dysfunction. It should also be noted that higher ketone levels are detrimental to vascular health, but moderate ketone levels protect against cardiovascular disease by improving insulin sensitivity and reducing inflammation in certain conditions [41,42,43,44,45].

Short-chain fatty acids (SCFAs), fatty acids of two to six carbon atoms produced by gut bacteria, play an important role in regulating inflammatory and oxidative stress pathways and mediators such as NF-κB and Nrf. SCFAs mitigate endothelial cell damage, reduce inflammation, improve endothelial function, and regulate lipid metabolism, thereby mitigating the development and progression of atherosclerotic plaques, contributing to the improvement of atherosclerosis [46]. Lu et al. [47], using a mice model, reported that SCFAs prevent HFD-induced obesity by regulating G protein-coupled receptors (GPCRs) and gut microbiota. Protection against HFD-induced obesity via SCFAs may also be mediated by a PPARγ-dependent switch from lipogenesis to fat oxidation [48]. Obesity is a risk factor for atherosclerosis; increased SCFAs attenuating HFD-induced obesity may be beneficial in atherosclerosis.

An HFD also contributes to adipose tissue deposition leading to weight gain and obesity [49], a risk factor for atherosclerosis. Adipose tissue deposition is also influenced by epigenetic regulation involving DNA methylation and histone modifications, which can either activate or silence genes responsible for fat cell development and function. Epigenetic changes in adipose tissue, potentially leading to increased fat accumulation, may be due to dietary habits, exercise levels, stress, and exposure to toxins which are also risk factors for atherosclerosis. The epigenetic changes in adipose tissue may contribute to increased inflammation and metabolic dysfunction associated with obesity-related diseases like type 2 diabetes via alteration in the expression of genes related to fat storage and metabolism expressed in adipose tissue [50,51,52]. This suggests that HFD-induced adipose tissue deposition leading to obesity and metabolic syndrome may be of therapeutic importance in personalized medicine by lifestyle modification.

## 4. High-Fat Diet, Epigenetics, and Cardiovascular Diseases

The risk of cardiovascular disease in offspring increases with maternal overfeeding and obesity during pregnancy and lactation, and this is due to abnormalities in cardiometabolism. A maternal high-fat diet increases the risk of the development of calcified atherosclerotic plaques in the offspring, involving phenotypic switching of vascular smooth muscle cells (VSMCs) to an osteochondrocytic-like phenotype [53]. A study fed a high-fat diet to 7-week-old female apo-E^−/−^ mice with a C57BL6/J background. The offspring were also fed a high-cholesterol diet after weaning. Histological findings supported the presence of calcified atherosclerotic plaques in the offspring at 3 months of age, and in vitro findings suggested a role of phosphate and IL-1β-induced osteochondrocytic transformation of VSMCs, contributing to plaque formation with a high-fat diet. A high-fat diet in pregnant mothers also results in the development of insulin resistance in the offspring, another contributor to atherosclerosis. The findings also support the role of inflammation in atherosclerotic plaque formation, progression, and vulnerability in the presence of hypercholesterolemia as documented by previous studies [9,11,14,54,55,56,57,58].

A high-fat diet is also associated with a change in gene expression and genome-wide DNA methylation, a mechanism contributing to epigenetic effects related to cardiovascular diseases. A high-fat diet causing obesity increases the risk of cardiovascular diseases, and Keleher et al. [59], using small (SM/J) mice, investigated the epigenetics of obesity induced by a high-fat diet and its effects on gene expression. The study found 4356 differentially expressed genes associated with a high-fat diet and 184 genes with a sex-by-diet interaction. The study found 7000 differentially methylated genes and multiple dysregulated pathways associated with high-fat diets. These findings suggest that a high-fat diet has an epigenetic effect on gene expressions involving methylation. The differential methylation and differential expression of ADAM metallopeptidase domain 11 (*Adam11*), UDP-N-acetyl-alpha-D-galactosamine polypeptide N-acetyl-galactosaminyltransferase 10 (*Galnt10*), ladinin 1 (*Lad1*), collagen type I alpha 1 chain (*Col1a1*), and ATP binding cassette subfamily G member 5 (*Abcg5*) with a high-fat diet [59] suggest its role in vascular disease. *ADAM11* regulating endothelial cells [60], *Galnt10* regulating intima-media thickness [61], and the involvement of *Col1a1* in plaque formation and vulnerability [12] suggest the role of a high-fat diet in vascular diseases involving epigenetics.

## 5. High-Fat Diet, Epigenetics, and Atherosclerosis

A high-fat diet contributes to atherosclerosis by increasing low-density lipoprotein (LDL) cholesterol, increasing foam cell formation, increasing endothelial lipase levels, altering the gut microbiota, increasing inflammation, increasing immune cell regulation, reprogramming neutrophils, and increasing the presence of fatty acids [5,21,62,63]. As discussed above, an HFD is associated with epigenetic changes, and the association between HFDs and atherosclerosis suggests the role of epigenetics in atherosclerosis. Studies have summarized and reported that plaque formation, progression, and vulnerability, vessel stenosis, vessel thrombosis, and the underlying molecular mechanisms involving inflammation are regulated by transcription factors, microRNA, and ncRNAs [10,13]. This suggests the role of epigenetics in the pathophysiology of atherosclerosis. Epigenetic changes are not associated with a change in DNA sequences, while genomic epigenetic status varies in a tissue-specific manner. The epigenome is the epigenetic status of a cell, and a change in the epigenome is due to methylation and histone modification [64].

Endothelial cell dysfunction plays a critical role in atherosclerosis. Studies have suggested that genetic variants of *NOS3*, *GUCY1A3*, *EDN1*, *PHACTR1*, *MFGE8*, *FLT1*, *JCAD*, *KLF2*, *PLPP3*, and *ARHGEF26* are associated with endothelial cell dysfunction and coronary artery disease [65]. Epigenetic mechanisms are associated with oxidative stress and inflammation, both playing a critical role in endothelial dysfunction and the pathogenesis of atherosclerosis. Epigenetic changes are also related to hemodynamic-mediated transcriptional changes and shear-stress-mediated plaque development. The upregulation of a catalytic component of the polycomb repressive complex 2 (*PRC2*) mediating tri-methylation of lysine 27 on histone 3 through EZH2; increased PRC2 methylating lysine 27 of histone H3 (H3K27me3) in endothelial cells isolated from atherosclerotic plaques; increased EZH2 expression in the endothelium in blood vessels in the regions of disturbed blood flow; and the role of acetylation and methylation in regulating *NRF2*, *KLF2*, *KLF3*, *KLF4*, and *HoxA5* suggest the role of epigenetics in atherosclerosis [64]. Further, the association of histone methyltransferase and demethylases such as EZH2 with foam cell formation; SIRT1 and SIRT 6 promoting cholesterol efflux; the association of histone acetyltransferase and deacetylases including P300, SIRT1, SIRT6, HDAC3, and HDAC9 with macrophages activation; HDAC4, SIRT2, JMJD3, PRMT1, and SMYD3 promoting and HDAC3 and HDAC9 opposing M2 macrophage polarization; and JMJD2D, SET7 UTX, HDAC1, and HDAC3 promoting and JMJD1A, SMYD2, SIRT1, and SIRT2 opposing M1 macrophage polarization suggest the role of epigenetics in regulating immune cells phenotype and function during atherosclerosis. The role of epigenetics in regulating various other immune cells has been discussed elsewhere [64]. Likewise, endothelial cells, immune cells, vascular smooth muscle cells, and fibroblasts also play a critical role in atherosclerosis, and this has been reviewed by Khan et al. and Jiang et al. [64,66]. Furthermore, the interaction between epigenetic factors and transcription factors including DNA hypermethylation with KLF4, as well as the interactions between DNA demethylation enzyme TET2 and RUNX1; DNMT3b and RUNX2; SETD7 and NF-κB; EZH2 and KLF2, KLF4, and STAT1; long noncoding RNA CDKN2BAS1 and EZH2 and CTCF; G9a and ATF7; HDACs and KLF2 and NRF2; and SIRT1 and NF-κB contribute to atherosclerosis [67]. The regulation of the expression of transcription factors due to a high-fat diet and atherosclerosis, like the activation of NF-κB, leads to increased secretion of inflammatory cytokine TNF-α, interferon-γ (IFN-γ), and inducible nitric oxide synthase (iNOS), suggesting that an HFD plays a critical role in atherosclerosis involving epigenetics [68,69].

The role of epigenetics in atherosclerosis is supported by Huang et al. reporting that epigenetically altered macrophages promote the development of atherosclerosis involving alterations in the epigenetic regulator histone deacetylase 3 (HDAC3) in the macrophages isolated from atherosclerotic arteries in human diabetic patients [70]. The associations between HDAC3 positivity and increased levels of LDL and triglycerides and decreased levels of HDL suggest the effects of dyslipidemia characterized by an HFD. Vascular smooth muscle cells acquire a unique cell-specific epigenetic signature during development and are not terminally differentiated. VSMCs undergo a phenotypic change during intimal injury, hyperlipidemia, and vascular disease, and modifications of the VSMC epigenetic programming are associated with SMC phenotype modulation (phenotypic switch) which is also regulated by interactions with the immune response [71,72]. Xu et al. [73] reported that the VSMC phenotypic switch and vascular remodeling are also regulated by SRF SUMOylation, an epigenetic process, and are induced by an HFD [74,75]. This suggests that the phenotypic switch of VSMCs, which is associated with a high-fat diet [76], involves epigenetic changes and contributes to atherosclerosis. Furthermore, the role of epigenetics in atherosclerosis is supported by the role of epigenetic mediators G9a, SUV39H1, and Set7 in regulating the gene expression of IL-6, IL-12p40, MIP-1α, MIP-1β, NF-κB p65, HOXA, FOXO, KLF4, IRF8, TNFα, IL-18, MCP-1, MMP9, MMP2, CD36, SR-A, and IRF8, and these interactions are involved with altered expression during the process of atherosclerosis, suggesting the role of epigenetics in atherosclerosis [66].

An HFD is associated with increased DNA methylation [22,59]; a study by Leung et al. reported on HFDs’ association with changes in chromatin accessibility in the mouse liver, and specific transcription factors and histone post-translational modifications are associated with these persistent chromatin accessibility changes after administration of an HFD [77]. This notion is further supported by the findings that an HFD initiates both reversible and persistent epigenetic changes if medaka fish (*Oryzias latipes*) are fed with an HFD in early life [78]. These studies were conducted on animal models. The studies in the human population, mainly with obese and diabetic subjects, suggest that an HFD increases DNA methylation and histone modification mainly in adipose tissue and the liver by increasing the availability of methyl donors contributing to epigenetic and metabolic changes leading to disease development. These epigenetic changes involve DNA methylation of leptin (*LEP*), adiponectin (*ADIPOQ*), insulin (*INS*), insulin receptor substrate 1 (*IRS1*), phosphatidylinositol 3-kinase regulatory subunit (*PIK3R1*), peroxisome proliferator-activated receptor γ coactivator 1 alpha (*PGC1A*), insulin-like growth factor 2 (*IGF-2*), Pro-opiomelanocortin (*POMC*), Neuropeptide Y (NPY), hypoxia-inducible factor 3a (*HIF3A*), tumor necrosis factor (*TNF*), interleukin 6 (*IL-6*), and mitochondrial transcription factor A (*TFAM*), while histone modification of CCAAT enhancer-binding protein β (*C/EBPB*), *C/EBPA*, preadipocyte factor-1 (*Pref-1*), adipocyte protein 2 (*aP2*), peroxisome proliferator-activated receptor γ (*PPARG*), POMC, and NPY mainly involve acetylation [7,8]. Studies have [8,79,80,81] summarized the role of ncRNA in regulating epigenetics in obesity, diabetes, and other chronic inflammatory diseases. This suggests that HFD-induced epigenetic changes are associated with the progression of atherosclerosis as well as the comorbid conditions and the risk factors for atherosclerosis. This notion is supported by the findings of high blood pressure and sustained increased levels of leptin in offspring through epigenetic memory in mothers fed with an HFD [82]. High blood pressure and increased leptin levels are risk factors for atherosclerosis [83,84].

Chronic inflammation in atherosclerosis is mainly driven by macrophages, and epigenetics plays a critical role in macrophage polarization by regulating DNA methylation or histone modifications. The skewing of macrophage polarization from M1 to M2 macrophages via epigenetic modifiers makes them an attractive therapeutic target. The role of epigenetics in atherosclerotic inflammation is supported by the involvement of alterations of DNA methylation involving DNA methyltransferases (DNMTs) and Ten-eleven translocation (TET) family proteins, methylation of lysine on histone and non-histone proteins by protein lysine methyltransferases (KMTs), and lysine demethylases in macrophage polarization in atherosclerosis-associated macrophages. Further, the involvement of histone methylation in trained immunity involving atherosclerosis, histone acetyltransferases in macrophage polarization, histone deacetylases in macrophage polarization and associated macrophages, and non-histone acetylation in macrophages highlights the role of epigenetics in atherosclerosis-associated inflammation [85]. Early intermittent hyperlipidemia contributes to altered tissue macrophages fueling atherosclerosis; however, the precise mechanisms of how it increases the risk of atherosclerosis-associated cardiovascular disease is not clear [86], and epigenetics may be an underlying denominator. This notion is supported by the finding of histone deacetylase 3 (HDAC3) as the most significantly altered epigenetic regulator in atherosclerotic lesion’s macrophages in human patients with diabetes and the attenuation of atherosclerotic plaques with depletion of HDAC3 in mice models. These findings support the role of epigenetically modified macrophages in atherosclerosis [70].

Macrophages are primary cells secreting cytokines in the pathogenesis of inflammation, and since macrophage polarization is regulated by epigenetics, cytokine expression must be epigenetically regulated. The cytokine levels may also be affected by changes in the macrophage phenotype which is regulated epigenetically, as discussed above. Epigenetic regulation of pro- and anti-inflammatory cytokines through DNA methylation and histone modifications plays a significant role in atherosclerosis. Epigenetics is associated with altered expression of inflammatory cytokines like TNF-α, IL-1α, and IL-6 and anti-inflammatory cytokine IL-10. For instance, increased DNA methylation at the TNF-α promoter can lead to higher expression of TNF-α, while hypomethylation at the IL-10 gene locus can promote IL-10 expression [85,87,88]. IL-1 and TNF-α are involved in the proliferation and differentiation of VSMCs, the activation of monocytes and macrophages, and the secretion of various inflammatory mediators in the pathogenesis of atherosclerosis [89,90]. Thus, targeting these cytokines has therapeutic potential, and a recent study using osteosarcoma cell lines revealed that TET enzymatic inhibition influences cell proliferation induced by inflammatory cytokines IL-1β and TNF-α, which have different effects on DNA demethylation [91]. These findings may be useful in suppressing inflammation and abnormal cell proliferation in atherosclerosis by modulating IL-6 expression levels and cell proliferation, though warranting research.

Senescent cells near the atherosclerotic lesion promote atherosclerosis by secreting various factors in concurrence with significant epigenetic alterations, and therapeutic removal of these senescent cells could delay or even prevent atherosclerosis. It is also evident that the accumulation of senescent cells promotes inflammation and negatively affects plaque remodeling [92,93]. The senescence-associated secretory phenotype (SASP) is a marker of cell (endothelial and VSMCs) senescence, which in turn is a vital source of the inflammatory response in atherosclerosis, making it an attractive therapeutic target [94]. The involvement of epigenomic changes mediating changes in cell function during vascular senescence [95] suggests the contributory as well as therapeutic role of epigenetics in atherosclerosis.

Oxidative stress promoted by a high-fat diet is another important factor contributing to atherosclerosis via endothelial damage, the accumulation of lipids in the arterial wall, and inflammation [96]. Oxidative stress induces epigenetic changes in the cells involved in plaque formation by altering gene expression leading to reprogramming of cells involved in cell proliferation, inflammation, and lipid metabolism via DNA methylation, histone modifications, and hypermethylation of genes involved in antioxidant defense. These epigenetic changes contribute to increased or decreased accessibility of chromatin to transcription factors, endothelial dysfunction, increased smooth muscle cell proliferation, macrophage activation, and chronic inflammation, all contributing to atherosclerosis. This makes epigenetic changes and mediators potential therapeutic targets in atherosclerosis [64,97,98,99,100]. Noncoding RNAs (ncRNAs), playing a critical role in the pathogenesis of atherosclerosis [13], are emerging as key regulators of this process via modulation of gene expression and can either promote or protect against oxidative stress in the vascular system. Modulation of gene expression by ncRNA is mediated by activating or repressing gene transcription post-transcriptionally or by chromatin remodeling by epigenetic processes. Furthermore, ncRNAs including long noncoding RNAs, micro RNAs, and circular RNAs are related to oxidative stress and reactive oxygen species production in atherosclerosis, contributing to endothelial dysfunction, macrophage activation and polarization, and vascular injuries [101]. Furthermore, the mutual regulation relationship between histone modifications and ncRNAs in atherosclerosis supports the role of epigenetics in the pathogenesis and the therapeutic avenues for targeting them to attenuate atherosclerosis progression [102]. For example, the ncRNA Chaer regulates H3K27me3 and interacts with the polycomb repressor complex (PRC) 2, promotes cell proliferation, and induces apoptosis in atherosclerosis involving mammalian target of rapamycin (mTOR) signaling [103]. The expression of antisense noncoding RNA in the INK4 locus (ANRIL) is associated with atherosclerosis, and the interaction of ANRIL with proteins of the PRC1 and PRC2 families leads to repressive H3K27me3 deposition on the target genes CDKN2A and CDKN2B, involving histone modification and chromatin remodeling [102]. Since epigenetics play a critical role in atherosclerosis, modulating epigenetic processes may be beneficial. Bioactive natural compounds such as omega-3 fatty acids, lycopene, or polyphenols including flavonoids, curcuminoids, and stilbenes acting through epigenetic mechanisms may have therapeutic potential by reducing inflammation, LDL cholesterol, and oxidative stress [104,105]. Dietary flavonoids, particularly anthocyanins, modulate inflammatory pathways through epigenetic mechanisms such as NF-κB signaling and Sirtuin-6 activation. Dietary intervention with flavonoids and polyphenols may modulate oxidative stress and inflammatory pathways, emphasizing the potential of bioactive compounds to counteract oxidative damage [106,107]. These findings emphasize how bioactive compounds in the diet may mitigate the adverse impacts of HFDs and counteract the harmful effects by modulating oxidative stress and inflammatory pathways, contributory factors in atherosclerosis.

## 6. Targeting Epigenetics in Atherosclerosis

Since epigenetic changes induced by HFDs play a critical role in the pathogenesis of atherosclerosis, targeting epigenetics and the factors regulating epigenetic changes should be potential therapeutic targets. Following a healthy lifestyle, a change in nutrition and exercise helps in decreasing the prevalence of cardiovascular diseases including atherosclerosis by modifying the epigenetic mechanisms. Modifying nutrition with a low-fat diet modifies DNA methylation, histone modification, and the gut microbiota with a beneficial effect on atherosclerosis [108]. Modifying nutrition to attenuate atherosclerosis is important in order to modify the metabolic pathways and cellular processes, because epigenetics modulate cardio-metabolic processes, and attenuating the prevalence of atherosclerosis by modifying these cardio-metabolic processes is important. The anti-atherogenic potential of polyunsaturated fatty acids (PUFAs) such as *n*-3 fatty acid (*n*-3 PUFA), α-linolenic acid (ALA), eicosapentaenoic acid (EPA), and docosahexaenoic acid (DHA) through vulnerable plaque stabilization, reduced platelet aggregation, decreased triglyceride levels, and attenuated inflammation has been suggested. The antioxidative effects of lycopene may protect against atherosclerosis, phytosterols significantly reduce LDL, and flavonoids present in tea, berries, cocoa, chocolate, and wine reduce the risk of CVD. Acting on the growth of atherosclerotic plaques, flavonoids decrease adhesion molecule expression, inflammation, and the capacity of macrophages to oxidate LDL, contributing to decreased atherogenesis through the modulation of methylation and histone modification [104,105]. Recently, Ji et al. [109] reported the anti-atherosclerotic potential of the bioactive compound Scutellariae Radix-Coptidis Rhizoma (QLYD) through multi-component, multi-target, and multi-pathway actions.

As mentioned above, hypercholesterolemia, hyperlipidemia, hypertriglyceridemia, folic acid, vitamin B, and other methyl donors are involved in epigenetic changes contributing to atherosclerosis; the inclusion of bioactive compounds like flavonoids, curcuminoids, and stilbenes (polyphenols) are cardioprotective through anti-inflammatory effects [110]. Studies suggest that exposure to obesogenic factors and diet, decreased physical activity and sedentary lifestyle, sleep deprivation, alcohol intake, weight loss interventions, and epigenetic drugs are associated with epigenetic changes. Modifying these factors and the use of DNMT inhibitors may be of therapeutic significance [8].

Therapeutic intervention to reverse abnormal epigenetic modification is possible because epigenetic modifications do not change the DNA sequence and are thus reversible, restoring the normal genome. Xu et al. [99] have summarized the role of epigenetic drugs, including the SIRT1 activators resveratrol, SRT1720, SRT2104, and SRT3025; the SIRT6 activators icariin, fucoidan, and cyanidin; the HDAC inhibitor SAHA; the DNMT inhibitor 5-aza-2′-deoxycytidine; natural DNMT inhibitors like quercetin and EGCC; natural inhibitors of CBP/p300 like curcumin; the EZH2 inhibitors like statins; the TET2 inhibitor vitamin C; HDAV inhibitors like valproic acid and phenylbutyrate; BET inhibitors like RVX-208 and +(-)JQ1; and the JMJD3 inhibitor GSKJ4, as well as their pharmacological actions and the model used for their therapeutic effects in atherosclerosis and associated vascular diseases. Preclinical and clinical trials targeting histone acetylation have shown promising results. HDAC3 and HDAC9 are promising targets, and apabetalone, a bromodomain and extraterminal (BET) protein inhibitor, attenuates inflammation, but its efficacy to attenuate atherosclerosis and cardiovascular diseases has not been established yet [111]. Targeting ncRNAs has also been suggested to be a promising therapy but needs more preclinical and clinical trials [99]. Epigenetic changes promoting atherosclerosis may also be targeted by modifying DNA methylation and via modulation of RNA gene expression [112]. Anti-epigenetic drugs including HDAC inhibitors (such as SAHA), 5-aza-2′-deoxycytidine, resveratrol–salicylate-targeting DNMTs, SIRT1, NF-κB, PPARγ, LOX, iNOS, procainamide, hydralazine, and others that modulate epigenetic processes [113,114] may be another avenue for personalized medicine in addition to nutritional intervention.

Macrophage polarization towards the M1 phenotype contributes to atherosclerosis through inflammation, while the M2 phenotype promotes anti-inflammatory cytokines. Further, macrophages are involved in foam cell formation, promoting atherosclerotic plaque formation. Thus, targeting macrophage polarization has been suggested as a therapeutic strategy to attenuate atherogenesis [115,116]. As mentioned above, macrophage polarization may be regulated epigenetically, and targeting epigenetically induced macrophage polarization may be of therapeutic significance. Jin et al. reported that targeting epigenetic modifiers to regulate and reprogram macrophage polarization in inflammatory atherosclerosis may have a therapeutic anti-inflammatory effect. Epigenetic modifiers including TET2, DMNT3A, HDAC3, HDAC9, HDAC7, JMJD3, and KDM4A in macrophages may be targeted to attenuate inflammation, but caution must be taken when targeting them as these factors are involved in various physiological functions in macrophages. Furthermore, targeting epigenetically induced metabolic rewiring and its interaction with epigenetic modifiers may also be an attractive therapeutic target to regulate macrophage polarization [117]. Targeting innate immune pathways and signaling for long-term epigenetic reprogramming is an attractive evolving strategy to attenuate atherosclerotic load and cardiovascular diseases. The CANTOS, COLCOT, and LoDoCo2 clinical trials have evidence on targeting innate immune pathways to attenuate the risk of cardiovascular diseases [118]. Although these strategies are in the developing phase, they provide promising hope for the treatment of atherosclerosis, because these approaches may result in the alteration of gene expression through epigenetic mechanisms resulting in modulation of the inflammatory response in plaques, potentially leading to plaque stabilization and reduced cardiovascular risk.

## 7. Conclusions

The existing literature on the association of a high-fat diet with atherosclerosis suggests a positive correlation. Furthermore, preclinical studies have provided evidence on the association of epigenetic changes with atherosclerosis in the presence of a high-fat diet. It was also evident that epigenetic changes occurring in the lifetime are not heritable, but the epigenetic changes occurring in utero due to pregnant mothers’ high-fat diet are heritable and may manifest in the next generations. Furthermore, therapeutic drugs modifying or inhibiting the epigenetic modifiers like HDACs and DNMTs may be useful in reversing the abnormal gene expression predisposing the body to atherosclerosis. Taken together, to avoid these issues, it is important to reduce saturated fatty acids in the diet and avoid risk factors to attenuate atherosclerosis development and progression.
